# Hyperpolarization-activated ion channels as targets for nitric oxide signalling in deep cerebellar nuclei

**DOI:** 10.1111/j.1460-9568.2010.07226.x

**Published:** 2010-06

**Authors:** Gary W Wilson, John Garthwaite

**Affiliations:** Wolfson Institute for Biomedical Research, University College LondonGower Street, London WC1E 6BT, UK

**Keywords:** cerebellum, cGMP, hyperpolarization-activated cyclic nucleotide-modulated cation channel, nitric oxide, rat

## Abstract

Most biological effects of nitric oxide (NO) in the brain are mediated by guanylyl cyclase-coupled NO receptors, whose activation results in increased intracellular cGMP levels. Apart from protein kinase activation little is known about subsequent cGMP signal transduction. In optic nerve axons, hyperpolarization-activated cyclic nucleotide-modulated cation (HCN) channels, which bind cGMP or cAMP directly, were recently suggested to be a target. The aim here was to test this possibility more directly. Neurones of the rat deep cerebellar nuclei were selected for this purpose, their suitability being attested by immunocytochemistry showing that the principal neurones expressed guanylyl cyclase protein and that NO synthase-containing fibres were abundant in the neuropil. Using whole-cell voltage-clamp recording, HCN channels in the neurones were activated in response to isoprenaline and exogenous cAMP but only occasionally did they respond to NO, although exogenous cGMP was routinely effective. With the less invasive sharp microelectrode recording technique, however, exogenous NO modulated the channels reproducibly, as measured by the size of the HCN channel-mediated voltage sag following hyperpolarization. Moreover, NO also blunted the subsequent rebound depolarizing potentials, consistent with it increasing the hyperpolarization-activated current. Optimizing the whole-cell solution to improve the functioning of NO-activated guanylyl cyclase failed to restore NO sensitivity. Minimizing cellular dialysis by using the perforated-patch technique, however, was successful. The results provide evidence that HCN channels are potential downstream mediators of NO signalling in deep cerebellar nuclei neurones and suggest that the more general importance of this transduction pathway may have been overlooked previously because of unsuitable recording methods.

## Introduction

Nitric oxide (NO) functions widely as a transmitter in the central and peripheral nervous systems, exerting its physiological effects by stimulating receptors having intrinsic guanylyl cyclase activity, thereby leading to cGMP accumulation in target cells (Garthwaite, 2008). cGMP can then engage a number of mechanisms, including cGMP-dependent protein kinases, phosphodiesterases and ion channels. One class of ion channel that is potentially modulated by cGMP is the hyperpolarization-activated cyclic nucleotide-modulated cation (HCN) channel, which generates an inward current [termed hyperpolarization-activated current (*I*_h_)] at membrane potentials negative to approximately −50 mV. *I*_h_ helps set the resting membrane potential and influences neuronal excitability, synaptic integration and other properties (Frere *et al.*, 2004). HCN channels are formulated from four subunits, of which two (HCN2 and 4) confer the most marked modulation by cyclic nucleotides. The binding of cyclic nucleotides shifts the voltage dependence to more positive potentials, thereby increasing the available *I*_h_ current and speeding up its activation kinetics.

cAMP is commonly regarded as the natural ligand for HCN channels because it has been found to be 10- to 60-fold more potent than cGMP (DiFrancesco & Tortora, 1991; Ludwig *et al.*, 1998; Zagotta *et al.*, 2003). Nevertheless, there is evidence from native neurones that the NO-cGMP pathway can also engage this target. For example, exogenous NO modified oscillatory activity in thalamocortical relay neurones by acting on HCN channels (Pape & Mager, 1992); in trigeminal motor and mesencephalic neurones, NO and cGMP reversibly depolarized the membrane and reduced the firing threshold by a presumed action on HCN channels (Abudara *et al.*, 2002; Pose *et al.*, 2003); the cGMP derivative 8-bromo-cGMP concentration-dependently shifted the HCN channel activation curve in sensory ganglia in the depolarized direction (Ingram & Williams, 1996); and, in the substantia gelatinosa, 8-bromo-cGMP or exogenous NO enhanced *I*_h_ (Kim *et al.*, 2005).

Most recently, studies on rat optic nerve found that NO coming from endothelial cells persistently depolarizes axons through cGMP acting on HCN channels (Garthwaite *et al.*, 2006). This finding represented the first evidence that endogenous NO can act through HCN channels in the central nervous system. In addition, the channels were found to be similarly sensitive to exogenous cAMP and cGMP derivatives, in agreement with a previous result from sensory ganglia (Ingram & Williams, 1996).

The aim of the present work was to test more directly if neuronal HCN channels serve as targets for the NO-cGMP pathway. For this purpose, neurones of the deep cerebellar nuclei (DCN) were selected based on evidence that they possess cAMP-sensitive HCN channels (Chen *et al.*, 2005) and that NO synthase and NO-activated guanylyl cyclase are found abundantly there (Rodrigo *et al.*, 1994; Ding *et al.*, 2004).

## Materials and methods

### Tissue preparation

All procedures were in accordance with regulations of, and approved by, the UK Home Office. Post-natal day 8–13 Sprague-Dawley rats (Charles Rivers, Ltd) were killed by cervical dislocation and decapitation. Juvenile rats were used because, beyond this age range, the greater extent of myelination diminishes both the visibility and viability of the neurones after slicing (Gauck & Jaeger, 2003). The brain was rapidly removed and the cerebellum dissected out, taking care to remove the meninges, and placed in iced (4°C) sucrose-substituted low Na^+^ artificial cerebrospinal fluid containing (in mm): 250 sucrose, 2.5 KCl, 6 MgCl_2_, 2.0 CaCl_2_, 1.0 NaH_2_PO_4_.H_2_O, 26.2 NaHCO_3_ and 11 glucose, equilibrated with 95% O_2_/5% CO_2_. Coronal slices (200–350 μm thick) were prepared in this solution using a vibroslicer and then maintained in a holding chamber containing artificial cerebrospinal fluid (119 mm NaCl, 2.5 mm KCl, 1.3 mm MgCl_2_, 2.0 mm CaCl_2_, 1.0 mm NaH_2_PO_4_.H_2_O, 26.2 mm NaHCO_3_ and 11 mm glucose equilibrated with 95% O_2_/5% CO_2_) first at 38°C for 1 h and at room temperature thereafter. The transient incubation at physiological temperature (20°C) was found to improve the yield of viable neurones located near the slice surface.

### Immunohistochemistry

Cerebellar slices containing the DCN or the whole cerebellum were fixed in ice-cold, freshly depolymerized paraformaldehyde (1%) in 0.1 m phosphate buffer (pH 7.4) for 2 h. Tissue was cryoprotected using ice-cold sucrose solution (5% for 3 h and then 20% overnight), then quickly frozen under dry ice in optimal cutting temperature embedding medium and sectioned coronally at 10 μm intervals onto chrome alum/gelatin-coated slides.

An immunoperoxidase staining procedure was applied to characterize the locations of neuronal NO synthase (nNOS) and NO receptor-associated guanylyl cyclases. A peroxidase suppressor in a methanol solution was used to inhibit endogenous peroxidase activity; this was applied for 15 min either before or after the primary antibody. The presence of NO-activated guanylyl cyclase was probed using two rabbit polyclonal antibodies. The first antibody was raised against a synthetic peptide corresponding to the rat β_1_ subunit, which is found in all known NO receptor isoforms [1 : 500; Cayman Chemical, MI, USA, 160897; peptide affinity-purified rabbit polyclonal anti-rat/bovine/human soluble guanylate cyclase β1 subunit raised against a synthetic peptide containing rat cyclase amino acids 188–207; suitable for immunohistochemistry as recommended by Ding *et al.* (2004)]. The second antibody was raised against a synthetic peptide corresponding to the rat α_1_ subunit [1 : 400; Sigma, Gillingham, UK, G4280; rabbit polyclonal anti-rat/human/murine/bovine soluble guanylate cyclase α_1_ subunit; suitable for immunohistochemistry as recommended by Makara *et al.* (2007)]. nNOS was also located using two antibodies: a rabbit anti-rat nNOS antibody [1 : 500; Zymed/Invitrogen 61-7000; epitope affinity-purified rabbit polyclonal anti-rat nNOS raised against a recombinant protein consisting of 195 amino acids from the N-terminal of rat nNOS protein; suitable for immunohistochemistry as recommended by Garthwaite *et al.* (2006)] and a sheep anti-rat nNOS antibody [1 : 10 000; a gift from Dr P. C. Emson, The Babraham Institute, Babraham, UK; suitable for immunohistochemistry as recommended by Garthwaite *et al.* (2006)].

Following rehydration of the slides with 0.1 m Tris-buffered saline containing 0.1% Triton-X-100, the tissue was blocked with 20% donkey serum and then incubated with primary antibody and 1% donkey serum overnight at 4°C. The slides were rinsed twice with the Tris-buffered saline/Triton-X-100 mixture and then once with Tris-buffered saline, and incubated with donkey anti-rabbit biotinylated secondary antibody for 1 h at room temperature. Slides were washed and incubated with Vectastain elite ABC (Vecto Labs Ltd, Peterborough, UK) for 45 min, stained for 4 min with 0.05% 3,3′-diaminobenzidine (Sigma, Dorset, UK) and then counterstained with Mayer’s haemalum for 15 s. Finally, slides were air-dried and mounted in distyrene, plasticizer and xylene medium and viewed using a Leitz Aristoplan microscope. All images presented are representative examples of sections from two or more animals. Brightfield photographs were adjusted using only the ‘auto levels’ and ‘auto contrast’ functions in Adobe Photoshop and the panels were then arranged in Adobe Illustrator.

### Electrophysiology

**Fig. 3:**
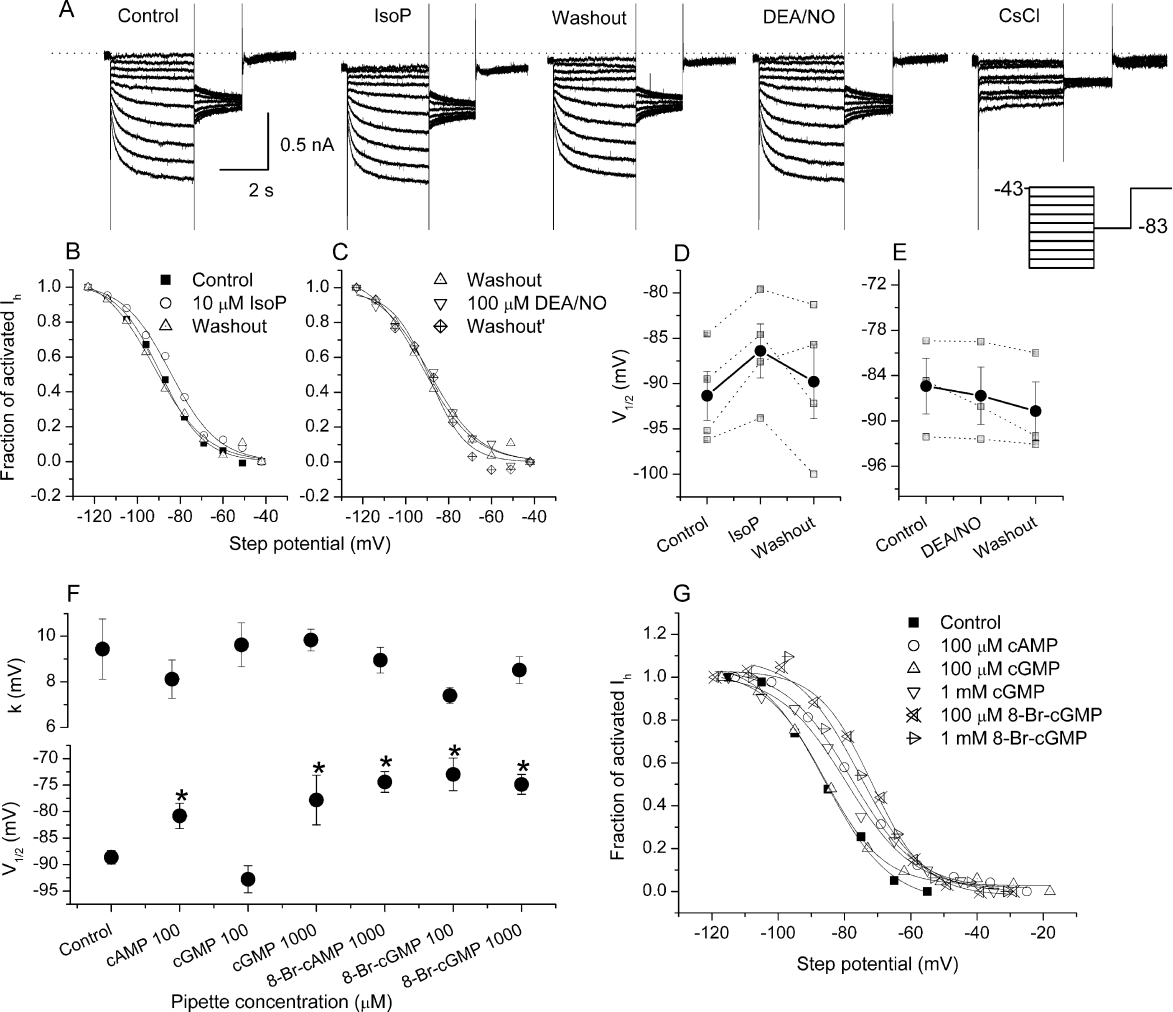
Modulation of HCN channels in DCN neurones by cyclic nucleotides in whole-cell recordings. (A) Sample traces showing that 10 μm isoprenaline (IsoP) caused a reversible, negative shift in the holding current, whereas 100 μm DEA/NO had no obvious effect and the HCN channel blocker CsCl inhibited the slowly activating inward current. The inset shows the voltage-clamp protocol, the holding potential being −43 mV. (B and C) Steady-state activation curves constructed from tail currents in A. (D and E) Summary data of the effect of 10 μm isoprenaline and 100 μm DEA/NO on *V*_1/2_ (*n* = 4 and 3, respectively; raw data in grey, means ± SEM in black). The shift in the presence of isoprenaline was significant (*t*_3_ = 4.66, *P* = 0.019), whereas the effect of DEA/NO was not (*t*_2_ = 1.19, *P* = 0.36). (F) A series of interleaved experiments showing the extent of modulation of HCN channel function by cyclic nucleotides added to the pipette solution. The data are expressed as the slope of the activation curves (k; top) and the *V*_1/2_ values (bottom). Each point is the mean ± SEM from two to five separate cells in different slices. *Significantly different from control by Student’s unpaired *t*-test (*P* < 0.03). k: 100 μm cAMP, *t*_8_ = 1.05, *P* = 0.32; 100 μm cGMP, *t*_7_ = 0.33, *P* = 0.75; 1 mm cGMP, *t*_6_ = 0.08, *P* = 0.94; 1 mm 8-bromo cAMP (8-Br-cAMP), *t*_7_ = 0.62, *P* = 0.55; 100 μm 8-bromo cGMP (8-Br-cGMP), *t*_5_ = 0.82, *P* = 0.45; 1 mm Br-cGMP, *t*_8_ = 0.84, *P* = 0.43. *V*_1/2_: 100 μm cAMP, *t*_8_ = 3.04, *P* = 0.016; 100 μm cGMP, *t*_7_ = 1.65, *P*=0.14; 1 mm cGMP, *t*_6_ = 2.76, *P* = 0.033; 1 mm 8-Br-cAMP, *t*_7_ = 5.93, *P* = 0.00058; 100 μm 8-Br-cGMP, *t*_5_ = 5.34, *P* = 0.0031; 1 mm 8-Br-cGMP, *t*_8_ = 6.10, *P* = 0.00029. (G) Representative steady-state activation curves for the conditions in F [the curve for 8-bromo cAMP (8-Br-cAMP) is omitted for clarity].

The slices were allowed to recover for at least 60 min and then one slice was transferred into a submerged slice recording chamber that was perfused continuously with artificial cerebrospinal fluid. All electrophysiological recordings were performed at 30–32°C. To study HCN channel function, 4-aminopyridine (4-AP) (1 mm) and tetrodotoxin (TTX) (500 nm) were present in the bath solution throughout; no additional voltage-dependent ion channel blockers were considered necessary because preliminary experiments showed that when CsCl (2 mm) was subsequently added to block HCN channels little active current remained (see Fig. 3A). Analysis was performed offline using Clampfit 8.0 (Axon Instruments, Foster City, CA, USA). For all recordings, ‘*n*’ refers to the number of individual cells, usually from different slices.

Whole-cell recordings were made from large-diameter (dentate or interposed) DCN neurones, visually identified using normal optics and a × 40 water-immersion objective (N.A .75, Zeiss). Pipette solutions initially contained (in mm): 150 KMeSO_4_, 10 KCl, 10 HEPES, 4 NaCl, 4 MgATP and 0.4 NaGTP. The pH was adjusted to 7.39–7.4 and the osmolarity to 280–290 mOsm/L. Open pipette resistance was 2–4 MΩ. Under voltage-clamp conditions, currents were recorded using an Axopatch 1D amplifier and pClamp7 (Axon Instruments) in neurones clamped at holding potentials from −40 to −55 mV. Resting membrane potentials (observed before and after TTX application) showed regular cycling between −37 and −68 mV. In all experiments, 80% compensation of the series resistance was applied and junction potentials were not corrected for. The recordings were filtered at 1 kHz and sampled at 2 kHz. The mean input resistance (*R*_input_) of the cells was 63 ± 8.6 MΩ (range 33–92; *n* = 7), similar to previous reports (Aizenman *et al.*, 2003). Patch longevity was usually a maximum of 30 min and, because of this limitation, CsCl (2 mm) was used to block *I*_h_ in preference to the organic blockers, such as ZD7288, which require a long time to exert their effect.

**Fig. 1:**
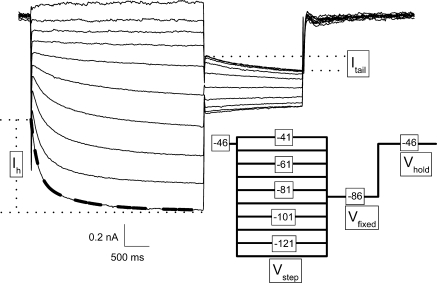
The *I*_h_ current in DCN neurones and its quantitation. A series of equal voltage steps lasting 3–4 s (*V*_step_) were applied (see inset), giving a family of currents, a typical one being illustrated in the main panel. Double exponential fits to the charging curves gave time constants for current activation (the fit for the most hyperpolarizing step is overlaid as a thicker dashed line). The voltage dependence of *I*_h_ was assessed by measuring the activation and deactivation tail currents upon return to a fixed potential (*V*_fixed_; approximately −85 mV in most cases); tail current amplitude (*I*_tail_) was measured as the difference between the current values at the beginning and end of this step. *V*_hold_ is the holding potential.

A series of long (3–4 s) voltage steps (*V*_step_) were used to analyse *I*_h_ (Fig. 1) and double exponential lines were fitted to the charging curve to give the fast and slow time constants for activation. The voltage dependence of *I*_h_ activation was assessed by measuring the activation and deactivation tail currents upon return to a fixed potential (approximately −85 mV for 2 s; *V*_fixed_ in Fig. 1) to remove the effect of the driving force; the tail current values were measured as the difference in current at the start and end of this voltage step. These current amplitudes were used to construct steady-state activation curves, which showed a typical S-shaped dependence on voltage and were fitted to the Boltzmann function. 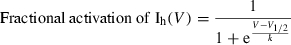
(1) where *V* is the membrane potential at *V*_step_, *V*_1/2_ is voltage at which the channels are the half-maximally activated and the slope factor, *k*, serves as an indication of the relation between voltage and fractional activation (see Banks *et al.*, 1993). Stably clamped cells often became leaky and unusable if prolonged hyperpolarizing voltage steps were repeatedly applied. Because of these technical limitations, we were unable to collect data at potentials where *I*_h_ was fully activated or deactivated. Therefore, the steady-state fractional activation function was obtained using shorter voltage steps and by normalizing the tail currents as follows
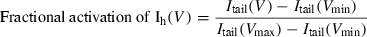
(2) where *V*_max_ and *V*_min_ are the voltages corresponding approximately to maximum and zero activation of *I*_h_, respectively.

On obtaining the whole-cell configuration, some cells demonstrated run-down in *V*_1/2_ values and so all recordings were allowed to stabilize over a 5–10 min period before experimental testing began. In order to minimize the effect of this unavoidable whole-cell phenomenon, paired *t*-tests were performed on control and test data. This means that a lack of observed change may be because variations in baseline *V*_1/2_ values could make it difficult to detect subtle changes.

**Fig. 6:**
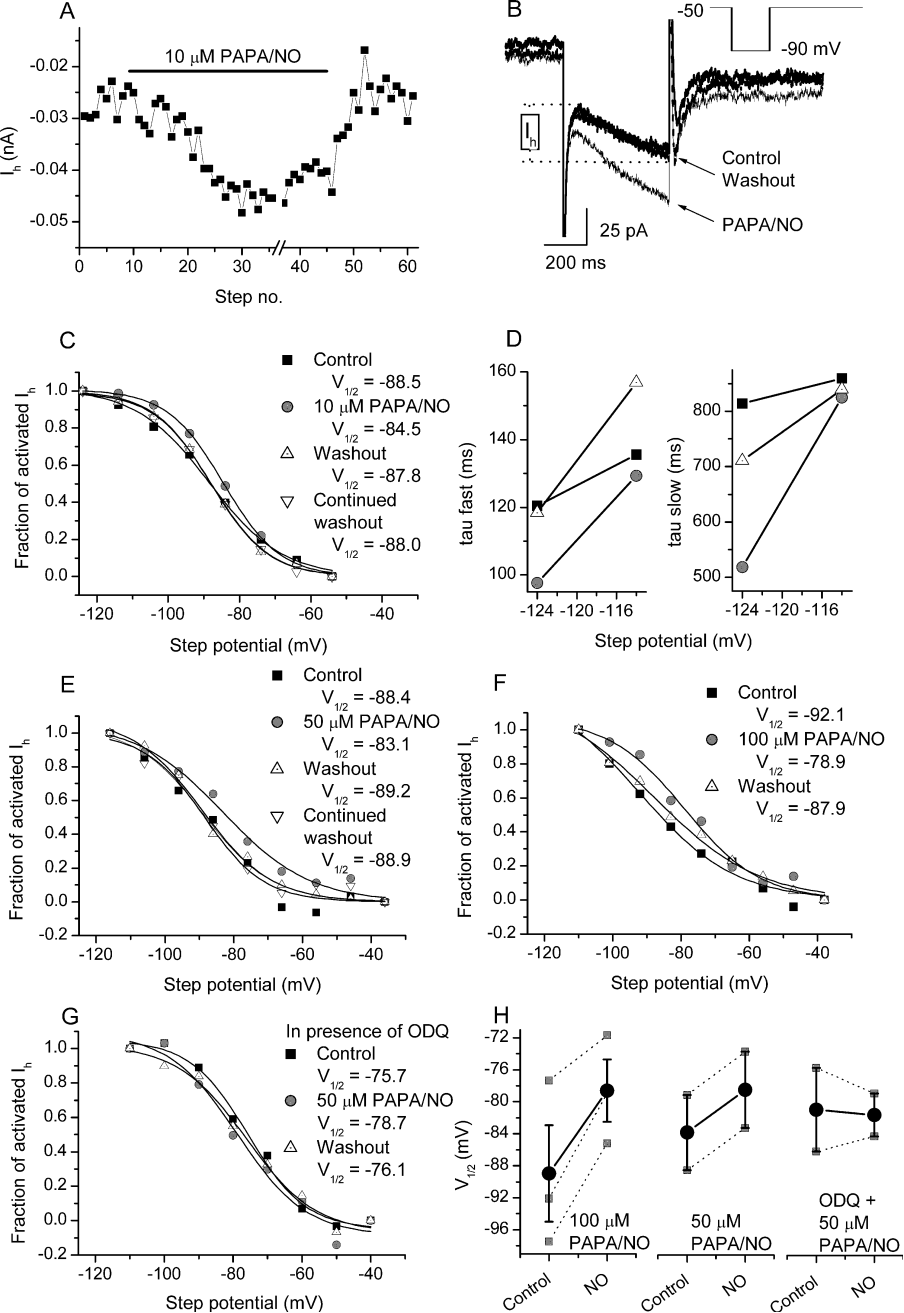
Voltage-clamp, perforated-patch recordings of the action of PAPA/NO on *I*_h_ in DCN neurones. (A) Example of the time-course of changes in the amplitude of *I*_h_ upon bath application and wash-out of 10 μm PAPA/NO. The break indicates when the protocol was applied for determining steady-state activation (see Fig. 1) in the presence of PAPA/NO. (B) Sample currents from the experiment in A; the protocol is shown in the inset. (C) Steady-state activation curves showing a reversible depolarizing shift in the voltage dependence of HCN channel activation in the presence of 10 μm PAPA/NO. (D) In the same cell as C, 10 μm PAPA/NO also caused a reversible speeding of the activation kinetics of *I*_h_ as determined by double exponential fits to the charging curves (see Fig. 1); legend is as for C. The effect of 50 μm (E) and 100 μm (F) PAPA/NO on the steady-state activation curves for *I*_h_ in different cells. (G) In the presence of 10 μm 1*H*-[1,2,4]oxadiazolo[4,3-a]quinoxalin-1-one (ODQ) throughout the recording, 50 μm PAPA/NO did not appear to affect the voltage dependence of activation for HCN channels in this cell. (H) Summary data showing the significant changes in *V*_1/2_ in the presence of 100 μm (*t*_2_ = 4.34, *P* = 0.049) and 50 μm (*t*_1_ = 107; *P* = 0.0060; non-directional test) PAPA/NO. In the presence of 10 μm ODQ, 50 μm PAPA/NO had no effect (*t*_1_ = 0.26, *P* = 0.84; non-directional test).

Test compounds were applied in the perfusate for 3–7 min, before the above step protocol was applied to study the properties of HCN channels. To follow the progress of wash-in (and wash-out), a small (approximately 40 mV), short (typically 500 ms) hyperpolarizing step (such as illustrated in Fig. 6A and B) from the holding potential was applied every 10 s to activate sufficient HCN channels to monitor changes. *I*_h_ was measured as the difference between the point following the capacitative transient and the plateau prior to the offset of the voltage step.

**Fig. 4:**
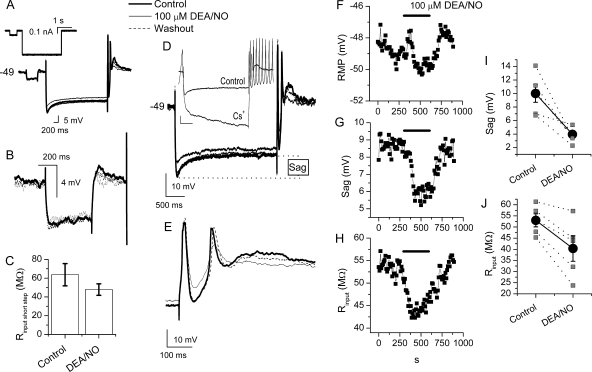
Current-clamp recordings using sharp electrodes of the effect of NO on *I*_h_ in DCN neurones. (A) Membrane potential response to hyperpolarizing current injections under control conditions (thick line), in the presence of 100 μm DEA/NO (thin line) and after wash of DEA/NO (dashed line); the protocol shown in the inset was delivered at 0.1 Hz. The resting membrane potential was −49 mV; traces are individual sweeps. (B) Magnification of the changes in membrane potential to the smaller, shorter initial current injection showing no apparent voltage sag or change in membrane properties in the presence or absence of NO because the voltage response falls at the foot of the steady-state *I*_h_ activation curve (see Fig. 1); traces are an average of five sweeps for each condition. (C) Summary of the passive input resistance (*R*_input short step_) calculated from data such as shown in B. The effect of DEA/NO was not significant (*t*_4_ = 2.32, *P* = 0.081). (D) Magnification of the response to the larger, longer current injection in A showing that 100 μm DEA/NO caused a reversible decrease in the depolarizing sag in the membrane potential. Inset: 2 mm CsCl inhibited the depolarizing sag and caused repeated spiking at the end of the hyperpolarizing step; the scales are the same as in the main panel. (E) The rebound voltage changes taking place on removal of the negative current step in D on a faster time-scale. Time-courses of the effect of 100 μm DEA/NO on the membrane potential (F), current being injected to maintain it at around −49 mV, on the amplitude of the depolarizing sag (G) and on the input resistance (H) during the long hyperpolarizing current injection. Summary of the changes in sag amplitude (I) and input resistance (J) in response to DEA/NO. Individual cells are represented by small squares and dotted lines; black circles are the means (± SEM). In I the results from one individual cell are hidden under the mean value. The effect of DEA/NO on both the sag (*t*_4_ = 4.12*, P*=0.015) and input resistance (*t*_4_ = 4.30, *P* = 0.013) was significant. All recordings were performed in the presence of 500 nm TTX and 1 mm 4-AP.

For sharp electrode recording, borosilicate microelectrodes (100–160 MΩ; Clark Capillaries, Reading, UK) were filled with 2 or 3 m potassium acetate. Blind recordings were made in either the lateral or interposed DCN. Upon entry into the cell, a strong hyperpolarizing current bias was applied to promote stability. The membrane voltage was filtered at 1 kHz and sampled at 2 kHz using an Axoclamp-2B amplifier (Axon Instruments) in bridge mode. The membrane potential was clamped at around −55 mV, as this potential was sufficiently hyperpolarizing to prevent spontaneous, rhythmic firing. The bridge balance was constantly monitored throughout the experiment and adjusted accordingly. The effect of a range of current injections was determined and the intensity adjusted to allow activation of *I*_h_. A step protocol (illustrated in Fig. 4) was applied every 10 s; a short (500 ms) and small hyperpolarizing step (insufficient to activate *I*_h_) was applied for the calculation of R_input short step_ when *I*_h_ is not activated and then a 3750 ms larger hyperpolarizing step to activate *I*_h_. The input resistance during this second step (*R*_input_) was determined using the average potential of the plateau that appeared following the voltage sag.

Gramicidin- and amphotericin-based perforated-patch recordings were also made from visually-identified large DCN neurones. The pipette solution contained (in mm): 150 KMeSO_4_, 10 KCl, 10 HEPES, 4 NaCl, 0.1 EGTA and 1 MgCl_2_. The pH was adjusted to 7.39–7.4 and the osmolarity to 280–290 mOsm/L. The open pipette resistance was 2–4 MΩ. A gramicidin stock solution was made in dimethylsulphoxide at 1 g/mL. Gramicidin or amphotericin was added to the pipette solution to a final concentration of 120–200 or 90 μg/mL, respectively, just before use and clarified through a 0.45 or 0.8 μm filter. Stable access resistances were obtained at 20–90 min after forming a gigaohm seal. If there was an abrupt increase in the amplitude of recorded current, signalling that the membrane had ruptured, the experiment was terminated. HCN channel function was investigated as for the whole-cell recordings.

### Assay of nitric oxide-activated guanylyl cyclase

Guanylyl cyclase activity was measured using a standard assay buffer as a benchmark (Griffiths *et al.*, 2003). Briefly, guanylyl cyclase purified from bovine lung (Axxora UK Ltd, Nottingham, UK) was incubated at 50 ng/mL in assay or test buffer (all containing 0.05 mg/mL bovine serum albumin), pre-warmed to 37°C, and exposed to 20 μm of the NO donor 2-(*N*,*N*-diethylamino)-diazenolate-2-oxide (DEA/NO) for 2 min. Samples were then inactivated and cGMP measured by radioimmunoassay. Three independent trials were carried out in each experiment. The standard assay buffer (pH 7.4) contained 50 mm Tris, 3 mm MgCl_2_, 0.1 mm EGTA, 0.05% bovine serum albumin and 1 mm GTP. Test solutions are described in Tables 2, 3 and 4. During the optimization of the whole-cell solution, the concentrations of free Mg^2+^and of Mg^2+^complexed with ATP, GTP and EGTA were calculated using the computer program Bound and Determined (Brooks & Storey, 1992).

**Table 2:** A Biochemical investigation of the effect of solution composition on NO-stimulated activity of purified guanylyl cyclase: Experiment 1

	Guanylyl cyclase activity (μmol/mg protein/2 min)
Assay buffer (3 mm Mg^2+^, 1 mm GTP)	16 ± 0.7
Assay buffer + KMeSO_4_ (150 mm)	15 ± 0.7 (*t*_4_ = 0.82; *P* = 0.46)
Assay buffer + KCl (10 mm)	17 ± 0.5 (*t*_4_ = −1.71; *P* = 0.16)
Assay buffer + HEPES (10 mm)	14 ± 0.4 (*t*_4_ = 2.68; *P* = 0.06)
Assay buffer + NaCl (4 mm)	18 ± 2.8 (*t*_4_ = −0.73; *P* = 0.51)

In Experiment 1, the individual whole-cell solution constituents were added separately to the reference assay buffer (50 mm Tris, 3 mm MgCl_2_, 0.1 mm EGTA and 1 mm GTP at pH 7.4) and had no significant detrimental effect on total cGMP produced on a 2 min exposure to 20 μm DEA/NO at 37°C.

**Table 3:** A Biochemical investigation of the effect of solution composition on NO-stimulated activity of purified guanylyl cyclase: Experiment 2

	Guanylyl cyclase activity (μmol/mg protein/2 min)
Assay buffer	19 ± 1.0
Whole-cell solution + 0 mm ATP	0 ± 0.4 (*t*_4_ = 18.11; *P* = 0.0005)
Whole-cell solution + 1 mm ATP	1 ± 0.3 (*t*_4_ = 17.74; *P* = 0.0006)
Whole-cell solution + 4 mm ATP	1 ± 0.4 (*t*_4_ = 16.74; *P* = 0.0007)

In Experiment 2, to try to replicate the whole-cell electrophysiological conditions in DCN neurones, the assay buffer was replaced with whole-cell solution (150 mm KMeSO_4_, 10 mm KCl, 10 mm HEPES, 4 mm NaCl, 0.4 mm GTP at pH 7.4) with or without the indicated ATP concentrations. Generation of cGMP was virtually abolished, irrespective of the ATP concentration, compared with the assay buffer.

**Table 4:** A Biochemical investigation of the effect of solution composition on NO-stimulated activity of purified guanylyl cyclase: Experiment 3

	Control (no ATP)	+ATP (1 mm)
Assay buffer	21 ± 3.2	–
Whole-cell solution + 0.6 mm Mg^2+^ + 0.4 mm GTP	2 ± 0.8 (*t*_4_ = 5.74; *P* = 0.005)	2 ± 0.6 (*t*_4_ = 5.96; *P* = 0.004)
+0.1 mm EGTA	17 ± 2.0 (*t*_4_ = 1.16; *P* = 0.31)	8 ± 0.8 (*t*_4_ = 4.00; *P* = 0.017)
Whole-cell solution + 3 mm Mg^2+^ + 1 mm GTP	7 ± 1.6 (*t*_4_ = 3.94; *P* = 0.017)	5 ± 0.2 (*t*_4_ = 5.10; *P* = 0.007)
+0.1 mm EGTA	15 ± 1.2 (*t*_4_ = 1.91; *P* = 0.13)	8 ± 0.6 (*t*_4_ = 4.19; *P* = 0.014)

In Experiment 3, the introduction of EGTA and free Mg^2+^ (at least 0.6 mm) restored guanylyl cyclase activity in the whole-cell solution. Two sets of Mg^2+^and GTP concentrations were tested; the first contained free Mg^2+^levels intermediate to the presumed physiological range (Grubbs, 2002) and the second had Mg^2+^and GTP concentrations similar to those in the assay buffer. Please note that the values for GTP are the total, final concentrations. All *P*-values represent a Student’s unpaired *t*-test between values for the assay buffer and the test condition; in all cases, *n* = 3.

### Materials

Forskolin and 1*H*-[1,2,4]oxadiazolo[4,3-a]quinoxalin-1-one were bought from Tocris Cookson (Bristol, UK), TTX from Latoxan (Rosans, France), KMeSO_4_ from City Chemical (CT, USA) and distyrene, plasticizer and xylene mounting medium and optimal cutting temperature embedding medium from VWR International (Dorset, UK); Mayer’s haemalum was purchased from Raymond A Lamb Ltd (Eastbourne, UK) and peroxidase suppressor from Pierce (Rockford, IL, USA). All other specialist chemicals were obtained from Sigma (Gillingham, UK). All standard reagents were obtained from BDH (VWR International). DEA/NO and (Z)-1-[*N*-(3-ammoniopropyl)-*N*-(*n*-propyl)-amino]diazen-1-ium-1,2-diolate (PAPA/NO) were obtained from Axxora UK Ltd. Stock solutions were dissolved in water except for forskolin, which was dissolved in dimethylsulphoxide (final concentration in artificial cerebrospinal fluid = 1%), and the NONOate NO donors, which were made up in 10 mm NaOH (final dilution at least 1 : 1000 into the bath solution).

### Statistics

Student’s paired and unpaired *t*-tests were conducted using Origin 6 or 7 (OriginLab Corporation, Northampton, MA, USA). Degrees of freedom were *n*−1 for paired measurements and (*n*_1_−1) + (*n*_2_−1) for independent measurements, and *P*-values < 0.05 were considered statistically significant. For sag amplitudes and input resistance, the mean of approximately five data points before and during the application of NO was used for statistical comparison. Data are presented as means ± SEM.

## Results

**Fig. 2:**
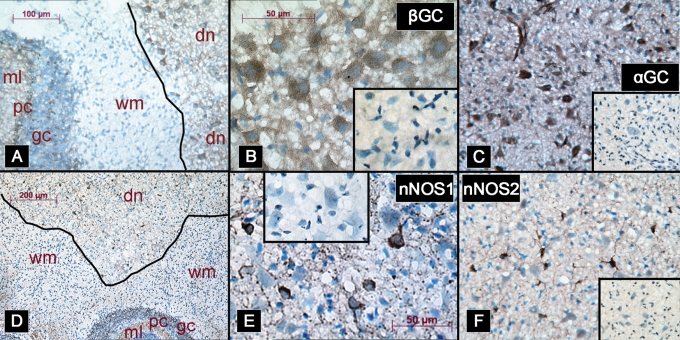
Immunocytochemical location of the proteins of the NO-cGMP pathway in the developing cerebellum. (A) The common β_1_-subunit of NO-activated guanylyl cyclase (βGC) (brown staining) appeared to be expressed in all layers of the cerebellar cortex and in the DCN, with little or none in the intervening white matter. The edge of the DCN is demarcated with a black line. (B) Higher magnification of part of A showing guanylyl cyclase staining throughout the cytoplasm of the majority of DCN neurones. (C) Staining for the α_1_-subunit of guanylyl cyclase (αGC) also showed a dense expression pattern; a blood vessel was stained in the top left-hand side of the field; scale is as for A. (D) Immunostaining for nNOS (brown) using the rabbit antibody was prominent in the molecular and granule cell layers of the cerebellar cortex, with little or no signal in the Purkinje cells or white matter, and there was moderate staining of the DCN, demarcated with a black line. (E) Higher magnification of part of D showing that, within the DCN, there was a distinct subset of nNOS-positive neurones and a network of stained varicose fibres throughout this region. (F) A sheep antibody raised against nNOS showed a distinct subset of stained neurones with processes ramifying throughout the DCN; scale is as for A. The insets (B, C, E and F) are controls incubated with secondary antibody only, showing no specific staining. dn, DCN; wm, white matter; ml, molecular layer; pc, Purkinje cell layer; gc, granule cell layer. All sections are counterstained with Mayer’s haemalum (blue).

Previous evidence had suggested that NO receptor-associated guanylyl cyclases (Gibb & Garthwaite, 2001; Ding *et al.*, 2004) and nNOS (Vincent & Kimura, 1992; Rodrigo *et al.*, 1994) are both present in the adult rat DCN. Because of technical limitations, our electrophysiological recordings were limited to developing, less myelinated tissue (Gauck & Jaeger, 2003) and so expression of the proteins in the juvenile DCN was first examined. Staining for the common β1-subunit of NO-activated guanylyl cyclases was obvious in all layers of the cerebellar cortex, whereas the white matter tracts located between the cortex and DCN were virtually devoid of staining (Fig. 2A). In contrast, the DCN showed strong staining in both the neuropil and the cytoplasm of the larger neurones (Fig. 2A and B). A similar pattern was seen when staining for the α_1_-subunit (Fig. 2C). Abundant nNOS staining (using the Zymed antibody) was observed in the granule cell and molecular layers of the cortex but was faint or absent in the somata of Purkinje cells (Fig. 2D). Unlike the cyclase, nNOS immunoreactivity was present in only a small number of cells in the DCN but there was an obvious network of nNOS-positive fibres ramifying throughout the nuclei (Fig. 2E). This pattern of staining in the DCN was also seen using a different antibody (Fig. 2F).

The presence of both components of the NO-cGMP signalling pathway in the DCN at the appropriate post-natal age, together with the presence of cyclic nucleotide-sensitive HCN channels in DCN neurones at a similar age (Chen *et al.*, 2005), allowed the hypothesis that NO-evoked cGMP signals modify neuronal function by engaging HCN channels to be tested directly. *I*_h_ is an unusual cationic channel that operates at quite hyperpolarized potentials and therefore, in different neuronal types, can contribute to resting membrane potential, length constant and dendritic integration (Robinson & Siegelbaum, 2003). In DCN neurones, *I*_h_ is active at potentials negative to approximately −50 mV (Chen *et al.*, 2005). To study the modulation of HCN channel function by NO, a series of long hyperpolarizing voltage steps (*V*_step_) to activate *I*_h_ were applied followed by a shorter step to a fixed potential (*V*_fixed_; Fig. 1). The tail currents generated upon return to *V*_fixed_ were normalized and plotted as a function of *V*_step_; these plots gave an indication of the voltage dependence of activation and the potential, *V*_1/2_, at which the channels were half-maximally activated (Fig. 3). As an initial control, we applied 10 μm isoprenaline, a β-adrenergic receptor agonist that classically acts by raising the levels of cAMP; this resulted in the expected positive shift in the voltage dependence of *I*_h_ activation (Fig. 3A, B and D). Perfusion of the NO donor DEA/NO (100 μm), however, had no significant effect (Fig. 3A, C and E). The control values for these neurones were −88.8 ± 2.34 mV for *V*_1/2_ and 210 ± 33 and 1099 ± 46 ms for the fast and slow activation time constants (*V*_step_ approximately −110 mV), respectively (*n* = 7). To probe cyclic nucleotide sensitivity more directly, cAMP or cGMP was included in the pipette solution. cAMP (100 μm) caused a depolarizing shift of approximately 10 mV, as did 1 mm (but not 100 μm) cGMP (Fig. 3F and G). The 8-bromo-substituted forms of the nucleotides, which are less prone to hydrolysis by phosphodiesterases (Wei *et al.*, 1998), were also effective, with 8-bromo-cGMP being maximally active at a concentration (100 μm) at which cGMP itself was inactive (Fig. 3F and G).

One explanation for cGMP but not NO being able to modulate the HCN channels could be that dialysis with the intracellular recording solution inhibits NO-evoked cGMP formation. Indeed, studies in this laboratory have found that the ATP concentration typically used in whole-cell recording solutions (4 mm) inhibits NO-activated guanylyl cyclase activity by about 90% (Roy *et al.*, 2008). Physiological ATP concentrations, by contrast, are around 1 mm (Gribble *et al.*, 2000), which causes much less (about 50%) inhibition (Ruiz-Stewart *et al.*, 2004). With 1 mm ATP in the whole-cell solution, 100 μm (*n* = 2 of 2), 10 μm (*n* = 4 of 6) or 1 μm (*n* = 5 of 5) DEA/NO had no effect on HCN channels. The *V*_1/2_ values in the latter two cases were −96 ± 4.4 (control) and −98 ± 3.7 mV (10 μm DEA/NO; *t*_3_ = 0.81, *P*=0.48), and −87 ± 2.2 (control) and −86 ± 2.4 mV (1 μm DEA/NO; *t*_4_ = 0.82, *P*=0.46). In some of these experiments, when patch integrity allowed, 1 mm 8-Br-cGMP (*n* = 2) or 1 μm forskolin (*n* = 1) was subsequently added by perfusion and an approximately 10 mV depolarizing shift in *V*_1/2_ was observed each time (data not shown). Moreover, it was notable that, in two out of the six recordings performed, 10 μm DEA/NO caused a depolarizing shift of similar magnitude that appeared to reverse on wash-out. The control *V*_1/2_ values were −88.6 and −93.5 mV, those in the presence of 10 μm DEA/NO were −84.6 and −80.3 mV, and after wash-out the values were −88.1 and −89.5 mV, respectively. Inspection of some of the properties of *I*_h_ in all cells exposed to 10 μm DEA/NO showed that there was no obvious difference in the resting membrane potential, *V*_1/2_, activation time constants or amplitude of *I*_h_ in those cells that responded compared with those that did not (Table 1).

**Table 1:** Comparison of some of the cellular and HCN channel properties for DCN neurones in which *I*_h_ was (cells 1 and 2) and was not (cells A–D) affected by 10 μm DEA/NO

			Time constants of activation (ms)		
Neurone	RMP (mV)	*V*_1/2_ (mV)	Slow	Fast	*I*_h_ amplitude (nA)
1	−44	−94	917	272	−0.19
2	−43	−89	1104	551	−0.18
A	−58	−90	1371	256	−0.30
B	−47	−90	1261	198	−0.17
C	−47	−98	1604	337	−0.15
D	−57	−108	1440	250	−0.21

The resting membrane potential (RMP) was measured at the beginning of each experiment. *V*_1/2_, the voltage at which the HCN channels were half-maximally activated, the amplitude of *I*_h_ and the activation time constants (when stepping to approximately −100 mV) were measured as described in Materials and methods and Fig. 1; all values were from control recordings prior to exposure to DEA/NO.

The variable response to NO with 1 mm ATP in the recording solution suggested that other ingredients of the electrode solution may also be detrimental. Accordingly, all components were examined systematically in a biochemical assay of cGMP formation by purified guanylyl cyclase (Tables 2, 3 and 4). The original whole-cell solution was found to be highly inhibitory to NO-evoked activity. Two factors were responsible: lack of EGTA, indicating an inhibitory effect of (background) Ca^2+^ (Kazerounian *et al.*, 2002), and free Mg^2+^, which is needed as a cofactor (Kimura *et al.*, 1976). The final ‘physiological’ whole-cell solution comprised (in mm): 150 KMeSO_4_, 10 KCl, 10 HEPES, 4 NaCl, 1 MgCl_2_, 0.1 EGTA, 1 MgATP and 0.4 NaGTP at pH 7.4 and 285 ± 5 mOsm/L. Despite success in the biochemical assay, the new solution failed to confer sensitivity of *I*_h_ in DCN neurones to exogenous NO, when the donor was either DEA/NO (50 μm; *n* = 2; *t*_1_ = 0.58, *P* = 0.67; non-directional test; data not shown) or a NONOate with slower release kinetics, PAPA/NO (100 μm; *t*_2_ = 1.01, *P* = 0.42; data not shown).

The possibility then arose that, for other reasons, the whole-cell recording technique disrupted NO signal transduction through the cGMP-HCN pathway in most of the neurones. This possibility was first tested using the less disruptive sharp microelectrode recording technique. Under current-clamp conditions, there was a depolarizing sag in the membrane voltage during a long, large hyperpolarizing step (Fig. 4A, D and I). After cessation of the hyperpolarizing step, there was a prominent rebound depolarization with one or more associated spikes (Fig. 4A, D and E) presumably caused by the activation of Ca^2+^channels (Aizenman & Linden, 1999) as TTX-sensitive Na^+^ channels were blocked. The addition of 2 mm Cs^+^ to block *I*_h_ removed the depolarizing sag (Fig. 4D, inset). Perfusion of 100 μm DEA/NO resulted in a decrease in the sag (Fig. 4A, D, G and I). The size of the depolarizing sag is an index of the degree of activation of *I*_h_, a decreased size implying that fewer HCN channels are available to be activated by the hyperpolarizing step because more were already active at the holding potential. During the application of NO, there was also a decrease in input resistance during this long hyperpolarizing current injection (*R*_input_; Fig. 4H and J), as expected from increased HCN channel activation. This interpretation was supported by a lack of NO-induced change in input resistance during a shorter, smaller step current when *I*_h_ is not activated (*R*_input short step_; Fig. 4A–C).

**Fig. 5:**
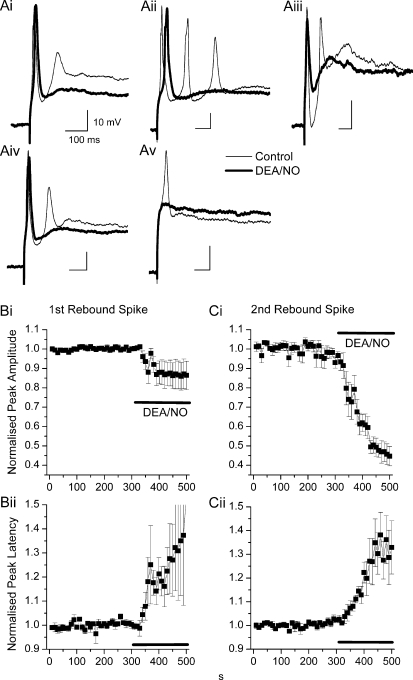
Sharp electrode, current-clamp recordings of the effect of NO on rebound potentials in DCN neurones. (A) The rebound potentials of all cells recorded (individually labelled as i–v). The plateau following the depolarizing sag was used as a baseline; scale bars are 100 ms and 10 mV. (B and C) Quantitative data for the rebound potentials in A. Values for the peak amplitude (Bi and Ci) and latency to peak (Bii and Cii) of the first (B) and second (C) rebound spikes are normalized to the mean values during the 300 s control period prior to addition of DEA/NO. Analysis by paired Student’s *t*-test of values at *t* = 0 and *t* = 490 s showed that DEA/NO caused non-significant changes in the amplitude and latency of the first spike (*t*_4_ = 2.02, *P*=0.11 and *t*_3_ = 1.32, *P* = 0.28, respectively) but significant changes in these parameters of the second spike (*t*_3_ = 8.46, *P* = 0.0035 and *t*_3_ = 2.91, *P* = 0.026, respectively). All recordings were performed in the presence of 500 nm TTX and 1 mm 4-AP.

There was also a change in the rebound depolarization in the presence of 100 μm DEA/NO (Figs 4A, D, E, and 5A, i–v). The first rebound spike of the depolarization showed a small, non-significant, decrease in amplitude and concomitant non-significant increase in latency to peak (Fig. 5B, i and ii). However, the second rebound spike was significantly smaller with a concomitant significant increase in latency to peak in the presence of NO (where it could be resolved; Fig. 5C, i and ii).

The positive results using sharp electrodes supported the hypothesis that normal whole-cell recording disrupts the signalling pathway. Accordingly, the next approach was to try the perforated-patch recording technique, which is much less invasive. Successful recordings over a sufficient period with this method, however, proved difficult. Nevertheless, using this method, NO (delivered using 10 μm PAPA/NO) was found to cause a reversible increase in the amplitude of *I*_h_ activated by a small hyperpolarizing step (Fig. 6A and B). This effect was attributable to a depolarizing shift in the voltage dependence of HCN channel activation (Fig. 6C). The rate of activation was also increased (Fig. 6D). In all cells (*n* = 6) exposed to PAPA/NO (10–100 μm) there was an apparent reversible enhancement of HCN channel function. In addition, the effect appeared to be NO concentration dependent in that cells exposed to 100 μm PAPA/NO (*n* = 3) showed a significant depolarizing shift in *V*_1/2_ of approximately 10 mV [Fig. 6F and H; control, −89.0 ± 6.0 mV vs PAPA/NO, −78.6 ± 3.9 mV], 50 μm PAPA/NO (*n* = 2) caused a shift of about 5 mV [Fig. 6E and H; control, −83.7 ± 4.7 mV vs PAPA/NO, −78.4 ± 4.8 mV] and with 10 μm PAPA/NO (*n* = 1) there was a 4 mV shift (Fig. 6C). To test if the observed effects of NO were via cGMP, 1*H*-[1,2,4]oxadiazolo[4,3-a]quinoxalin-1-one, the standard blocker of guanylyl cyclase-coupled NO receptors, was used. In two cells (out of a total of approximately 20 attempts) in which the experiment could be performed, PAPA/NO (50 μm) had no observable effect on the steady-state activation curve for *I*_h_ in the presence of 10 μm 1*H*-[1,2,4]oxadiazolo[4,3-a]quinoxalin-1-one [Fig. 6G and H; control, −80.9 ± 5.2 mV vs PAPA/NO, −81.5 ± 2.7 mV].

## Discussion

Given the evidence that endogenous NO regulates axonal function in the optic nerve through cGMP and a subsequent activation of HCN channels (Garthwaite *et al.*, 2006), the present investigation aimed to test directly if HCN channels may be considered more general targets for NO-cGMP signalling in neurones, bearing in mind that the channels are commonly viewed as being modulated primarily by cAMP (DiFrancesco & Tortora, 1991; Ludwig *et al.*, 1998; Zagotta *et al.*, 2003).

Addressing this straightforward aim proved frustrating but we can arrive at two main conclusions. The first is that the usual whole-cell recording technique can obliterate the signalling pathway under study. This was not occurring at the level of the HCN channels, which remained active and modifiable by cyclic nucleotides. One problem was the composition of the electrode solution, which was highly inhibitory to NO activation of guanylyl cyclase because of the abnormally high amount of ATP, lack of Ca^2+^ buffering and lack of excess Mg^2+^. As the solution is a conventional one for whole-cell recording from neurones and other cells, it remains possible that its use effectively eliminates a widespread cell–cell signalling pathway, regardless of the precise downstream mechanism. Nevertheless, despite the problems with the electrode solution, it transpired that the over-riding factor was the whole-cell recording technique itself. In this respect, it may be relevant that most previous publications dealing with NO/cGMP regulation of HCN channels in central neurones have used less disruptive sharp electrodes (Pape & Mager, 1992; Abudara *et al.*, 2002; Pose *et al.*, 2003). An exception was in substantia gelatinosa neurones where *I*_h_ was shown to be positively regulated by an NO donor and 8-bromo-cGMP under whole-cell voltage-clamp conditions (Kim *et al.*, 2005). Similarly, NO was able to shift the activation curve of HCN channels in the positive direction in sino-atrial nodal cells using a whole-cell solution of similar composition to our ‘physiological’ solution (Barbuti *et al.*, 2004). The reason why whole-cell recording is disruptive in some cells and not others remains obscure but may be related to the location of the NO transduction unit relative to the recording electrode.

The second conclusion from the recordings from DCN neurones is that HCN channels may be regarded as targets for NO released from either capillary endothelial cells (Garthwaite *et al.*, 2006) or nerve fibres in close juxtaposition (Fig. 2E and F); studies to test these possibilities are in progress. A limitation of the investigation (along with all others performed to date) is that it cannot be excluded that the effect of NO is indirect, resulting from the NO-dependent release of another mediator whose action is transduced through HCN channels. We attempted to address this issue by recording from neurones freshly isolated from the DCN using the method of Simeone *et al.* (2005) but, in all cells tested (*n* = 6), no *I*_h_ was visible, although voltage-dependent Na^+^ and K^+^ currents were clearly seen (not illustrated). Presumably, the channels are located on the more distal processes that are shorn off during the isolation procedure, an interpretation consistent with immunocytochemical findings that the HCN channel protein in the DCN is concentrated in the neuropil (Notomi & Shigemoto, 2004). Nevertheless, studies on isolated heart pacemaker cells have shown that NO (through cGMP) can directly affect HCN channels (Herring *et al.*, 2001). The lack of HCN channels in the isolated neurones was also disadvantageous from another perspective, in that it would have allowed the delivery of known NO concentrations directly to the cells under study to discover the levels required to engage this pathway. The studies using intact brain slices give no direct information on this point because NO is inactivated by the tissue extremely rapidly (predicted half-life of < 10 ms at 10 nm NO and below), which means that very steep NO gradients will exist across the tissue and therefore that the concentration experienced by a given neurone will depend on its position (Hall & Garthwaite, 2006). Hence, this remains an outstanding issue, and not only for neuronal channels. All that may be stated is that, based on the relative electrophysiological effects of PAPA/NO and DEA/NO in hippocampal slices, the NO concentrations activating *I*_h_ in the perforated-patch recordings using PAPA/NO (10–100 μm) as the donor are similar to those required to substitute for endogenous NO in synaptic plasticity (Bon & Garthwaite, 2001; Hopper & Garthwaite, 2006) and so are likely to approximate to physiological NO concentrations.

The molecular make-up of the HCN channels operating in the DCN neurones remains unclear. At the mRNA level, HCN2 and HCN4 are highly expressed in the nuclei compared with HCN1 (Ludwig *et al.*, 1998; Monteggia *et al.*, 2000; Santoro *et al.*, 2000; Chen *et al.*, 2005), whereas immunohistochemistry suggests the presence of protein for all three subunits (Notomi & Shigemoto, 2004). The mean *V*_1/2_ values and time constants in our study are similar to native channels in thalamocortical and hippocampal neurones that express different combinations of HCN1, 2 and 4 subunits (Santoro *et al.*, 2000; Surges *et al.*, 2006); the *V*_1/2_ values are intermediate to values reported previously for homomeric HCN2 and HCN4 channels (Accili *et al.*, 2002). Potentially, therefore, heteromeric channels underlie the currents in DCN neurones. Although HCN channels are commonly regarded as being regulated by the more potent cyclic nucleotide, cAMP, the responsiveness of DCN neuronal channels to NO may reflect the presence of guanylyl cyclase in the vicinity of the channels, producing cGMP in the low micromolar range needed to be effective (DiFrancesco & Tortora,1991; Ludwig *et al.*, 1998; Zagotta *et al.*, 2003; Zhou *et al.*, 2004). Such a possibility appears feasible as guanylyl cyclases in cells can generate up to 100 μm cGMP/s in response to NO (Mo *et al.*, 2004). Alternatively, some native channels may be less discriminating, considering evidence from the optic nerve and sensory ganglia that the 8-bromo derivatives of cAMP and cGMP were similarly potent HCN channel agonists when applied extracellularly (Ingram & Williams, 1996; Garthwaite *et al.*, 2006).

One functional outcome of applying NO to the DCN neurones was a clear reduction in the rebound depolarizations occurring post-hyperpolarization. As TTX-sensitive Na^+^ channels were blocked, these depolarizations are presumably carried by Ca^2+^channels. These could be low-threshold T-type Ca^2+^channels (Alvina *et al.*, 2009) that become deinactivated as a result of the hyperpolarization. However, it should be noted that the role of T-type channels in rebound spiking has recently been questioned when the depth of hyperopolarization does not extend beyond the chloride reversal potential; it may be that different types of Ca^2+^channels and/or modulatory mechanisms are involved (Zheng & Raman, 2009a; reviewed in Zheng & Raman, 2009b). Furthermore, it cannot be excluded that the decrease in the peaks could be another effect of NO. For example, there is some evidence in the literature of the acute inhibition of other (L-type) Ca^2+^channels by cGMP and cGMP-dependent kinase (Quignard *et al.*, 1997; Liu *et al.*, 1997). Information on the effects of cGMP on T-type channels is sparse, however, and relates to transcriptional regulation (Zeng *et al.*, 2005). A simpler explanation is that the changes in rebound depolarization in the presence of NO are secondary to changes in *I*_h_ because this current contributes importantly to the rebound depolarization and associated activation of T-type currents in these (Aizenman & Linden, 1999) and other (McCormick & Pape, 1990b) neurones. By analogy with the effect of noradrenaline on thalamic neurones (McCormick & Pape, 1990a), when *I*_h_ is enhanced, in this case by NO, the reduced hyperpolarization and increased membrane conductance (Fig. 4) together serve to inhibit T-type channel activation and, hence, inhibit the rebound spiking (Fig. 5). In DCN neurones, rebound spiking is particularly prominent following repeated stimulation of the inhibitory (GABAergic) Purkinje cell input and, by modifying the firing pattern of the neurones depending on the type of excitatory input to Purkinje cells, the rebound depolarization has been hypothesized to play a role in motor behaviour (Aizenman & Linden, 1999). Our results suggest a locus for the modulation of such behaviour by NO. A caveat to this interpretation of *in-vitro* data is that recent findings have questioned the importance of the rebound spiking in DCN neurones *in vivo* (Alvina *et al.*, 2008).

The degree of rebound depolarization may also have repercussions for synaptic plasticity between Purkinje cell axons and DCN neurones, in that a strong depolarization associated with high-frequency firing of action potentials tends to generate long-term potentiation, whereas a suppression of this phase produces long-term depression (Aizenman *et al.*, 1998). The rebound depolarization also appears to play a central role in governing long-term potentiation of excitatory inputs to the DCN neurones (Pugh & Raman, 2006). Combined with our results, this leads to the speculation that the presence of NO would favour long-term depression of Purkinje cell axon synapses and a suppression of long-term potentiation at excitatory synapses. Hence, by modulating HCN channels that help shape the post-hyperpolarization changes, the NO-cGMP pathway may play an important role in modulating synaptic plasticity in the DCN and thereby contribute to certain types of motor learning. For example, it has been shown that decreases in endogenous NO in the interposed nucleus of the DCN delayed classical eyelid conditioning (Allen & Steinmetz, 1996).

The generality of our findings carried out using the DCN remains to be investigated but it is noteworthy that cyclic nucleotide-sensitive HCN channels are widely distributed throughout the central nervous system (Notomi & Shigemoto, 2004) and signal transduction through the channels could help explain a number of observations of NO acting to modify neuronal membrane potentials or excitability and, potentially, synaptic plasticity (Garthwaite, 2008). An interaction with HCN channels may also contribute to the participation of the NO-cGMP pathway in certain clinical conditions, such as pain (Levy & Zochodne, 2004; Jiang *et al.*, 2008).

## Abbreviations

DCN: deep cerebellar nuclei
DEA/NO: 2-(*N*,*N*-diethylamino)-diazenolate-2-oxide
HCN: hyperpolarization-activated cyclic nucleotide-modulated cation
*I*_h_: hyperpolarization-activated current
nNOS: neuronal nitric oxide synthase
NO: nitric oxide
PAPA/NO: (Z)-1-[*N*-(3-ammoniopropyl)-*N*-(*n*-propyl)-amino]diazen-1-ium-1,2-diolate
TTX: tetrodotoxin
